# Functional ultrasound imaging of deep visual cortex in awake nonhuman primates

**DOI:** 10.1073/pnas.1916787117

**Published:** 2020-06-08

**Authors:** Kévin Blaize, Fabrice Arcizet, Marc Gesnik, Harry Ahnine, Ulisse Ferrari, Thomas Deffieux, Pierre Pouget, Frédéric Chavane, Mathias Fink, José-Alain Sahel, Mickael Tanter, Serge Picaud

**Affiliations:** ^a^Sorbonne Université, INSERM, CNRS, Institut de la Vision, F-75012 Paris, France;; ^b^Physics for Medicine Paris, INSERM, CNRS, École Supérieure de Physique et de Chimie Industrielles (ESPCI Paris), Paris Sciences et Lettes (PSL) Research University, 75012 Paris, France;; ^c^INSERM 1127, CNRS 7225, Institut du Cerveau et de la Moelle Épinière, Sorbonne Université, 75013 Paris, France;; ^d^Institut de Neurosciences de la Timone, UMR 7289 Centre National de la Recherche Scientifique and Aix-Marseille Université, 13385 Marseille Cedex 05, France;; ^e^Institut Langevin, CNRS, École Supérieure de Physique et de Chimie Industrielles (ESPCI Paris), Paris Sciences et Lettes (PSL) Research University, 75012 Paris, France;; ^f^Department of Ophthalmology, The University of Pittsburgh School of Medicine, Pittsburgh, PA 15213, United States;; ^g^Department of Ophthalmology and Vitreo-Retinal Diseases, Fondation Ophtalmologique Rothschild, F-75019 Paris, France

**Keywords:** visual cortex, nonhuman primate, functional ultrasound imaging, ocular dominance, brain imaging

## Abstract

Deep regions of the brain are not easily accessible to investigation at the mesoscale level in awake animals or humans. We have recently developed a functional ultrasound (fUS) technique that enables imaging hemodynamic responses to visual tasks. Using fUS imaging on two awake nonhuman primates performing a passive fixation task, we constructed retinotopic maps at depth in the visual cortex (V1, V2, and V3) in the calcarine and lunate sulci. The maps could be acquired in a single-hour session with relatively few presentations of the stimuli. The spatial resolution of the technology is illustrated by mapping patterns similar to ocular dominance (OD) columns within superficial and deep layers of the primary visual cortex. These acquisitions using fUS suggested that OD selectivity is mostly present in layer IV but with extensions into layers II/III and V. This imaging technology provides a new mesoscale approach to the mapping of brain activity at high spatiotemporal resolution in awake subjects within the whole depth of the cortex.

## Significance

Nowadays, several techniques exist to study and better understand how the brain works (fMRI, EEG, electrophysiology, etc.). Each has its own advantages and disadvantages (spatiotemporal resolution, maximal recording depth, signal-to-noise ratio, etc.). In this article, we show that the new functional ultrasound (fUS) imaging technique is appropriate to record and map brain activity in awake primates on a scale previously unreachable. It allows distinguishing patterns similar to ocular dominance bands in the visual cortex through all layers of the cortex, which was impossible before with common techniques. This paper demonstrates the utility of fUS imaging for studying brain activity in awake primates and its interest to all neuroscientists.

The visual system offers ideal terrain for the evaluation of novel technologies to measure and map brain activity in large mammals, because it embraces a large fraction of the cerebral cortex (1). Thanks to the study of very controlled visual stimulations in the visual field, the system is already highly characterized. For neuroscientists interested in vision, one fundamental set of objectives concerns the identification of the overall architecture of the visual cortex at mesoscale, while investigation of the neuronal computations that underlie behavior is pursued at microscale. Recent advances in neurotechnology have provided insights into these neuronal computations at the microcircuit level in rodents (2–4) and at the macrocircuit level in nonhuman primates using functional magnetic resonance imaging (fMRI) (5–7). However, a combined macro and micro approach to uncovering activity maps during behavior tasks is necessary to achieve a system-level understanding of visual information processing.

fMRI in visual cortex studies has yielded low-resolution retinotopic maps but ocular dominance bands were too small to be resolved in primates (8–10). Optical imaging provides micro- to mesoscopic resolution in nonhuman primates using either intrinsic optical imaging (11–15), voltage-sensitive dye imaging (VSDI) (16–21), or two-photon microscopy using calcium sensors (22–24). However, all three approaches have limited imaging depth, being either restricted to the cortical surface (optical imaging, VSDI) or to depths of only ∼600 μm (two-photon microscopy). The latter is also constrained by a restricted field of view (25, 26). This means that the deep cortical folds of the visual cortex within the calcarine sulcus are inaccessible to these techniques (27, 28). New functional ultrasound (fUS) imaging techniques have recently been found to provide high spatiotemporal resolution (100 μm, 1 Hz) even in deep structures (up to 1.5 cm) (29, 30), and have been applied to three-dimensional (3D) mapping of the visual system of rodents and pigeons (4, 29, 31). The technique measures changes in the cerebral blood volume (CBV) within microvessels using ultrafast Doppler (32), combined with spatiotemporal clutter filtering based on singular-value decomposition (33). Like fMRI, therefore, it relies on the neurovascular coupling of brain activity.

While early reports were based on studies in anesthetized animals (30, 34–36), more recent studies have involved awake rodents (4, 37–39) and awake primates performing tasks (40). Recent studies have demonstrated the capability of fUS imaging for high-resolution mapping of 3D tonotopic organization in the auditory cortex and deeper structures such as the inferior colliculus in awake ferrets (41), in neonates through the transfontanellar window (42), and perioperatively during tumor resection in adults (43).

In this study, we evaluate the reliability of fUS imaging for mapping the functional organization of the visual cortex, such as retinotopic maps and ocular dominance columns, in awake primates with an accuracy unreachable before. Despite noise factors, these preliminary results illustrate that the spatiotemporal resolution and sensitivity of fUS in deep tissues offer access to activity in nonoptically accessible deep cortical layers of the primary visual cortex as well as the even deeper cerebral areas along the calcarine and lunate sulci.

## Results

### Ten 0.5-s-Long Stimulations Are Sufficient to Map Cortical Activation.

**Fig. 1.**
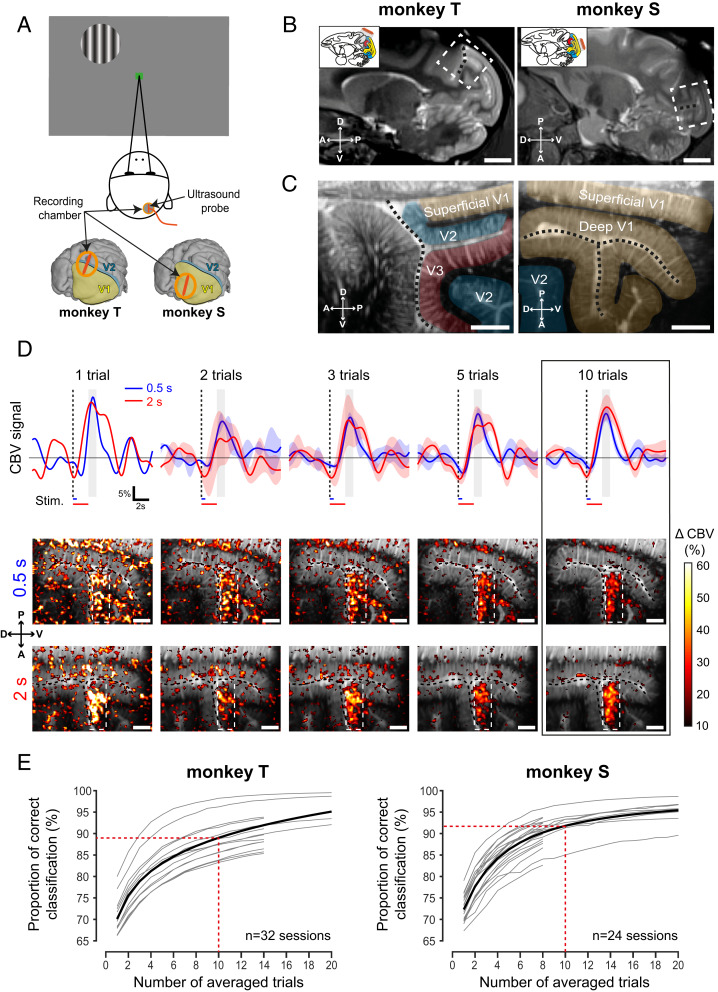
Optimization of the stimulation duration and the number of repetitions for fUS imaging. (*A*) Passive fixation task. While fixating a central green square, a peripheral visual stimulus was flashed for 0.5 or 2 s. The recording chamber (orange) was positioned above the right visual cortex in both animals. The US probe (deep orange) was sagittally oriented in this representation. (*B*) Anatomical MRI (ML +7 mm) showing the localization of the fUS imaging planes (white dotted rectangles) for monkey T (*Left*) and monkey S (*Right*). Lunate and calcarine sulci are, respectively, highlighted for monkey T (*Left*) and S (*Right*) with black dotted lines. (*B*, *Insets*) V1, V2, and V3 are, respectively, represented in yellow, blue, and red. The US probe (deep orange) and the corresponding in-depth imaging planes (gray) are schematized. (*C*) Deep anatomical fUS acquisition. These are the same sagittal slices as in Figs. 2 and 3 *A* and *B*. The black and white images represent the amplitude of the mean CBV. The different visual areas are represented by colors (yellow, V1; blue, V2; red, V3). The positions were determined by an atlas. (*D*) CBV measurements during repeated visual stimulation. (*D*, *Top*) Mean CBV signal variations within the ROI are represented in the maps (*Middle* and *Bottom*) by white dotted rectangles with an increasing number of trials for the two stimulation durations (blue, 0.5; red, 2 s; shaded bars, SEM). (*D*, *Middle* and *Bottom*) Activation maps obtained for 0.5 s (*Middle*) or 2 s (*Bottom*) of stimulation for the number of averaged trials (1, 2, 3, 5, and 10 trials). Black dotted lines, calcarine sulcus. (*E*) Statistical analysis of the measurements showing the proportion of pixels correctly classified with respect to an image generated with 20 other trials (32 sessions for monkey T; 24 sessions for monkey S) with 0.5-s stimulation duration. Gray lines represent the evolution for each individual session (sessions composed of only one trial are not plotted). Black lines represent the Naka–Rushton fitting result for all of the sessions. (Scale bars, 1 cm [*B*] and 2 mm [*C* and *D*].)

We recorded the variations of CBV using fUS in two monkeys while they were performing a passive fixation task (Fig. 1*A*). The recording chambers on the two animals were positioned on different but juxtaposed areas to have two fUS sagittal imaging planes contiguous on the visual cortex (medial-lateral +7 mm) (Fig. 1*B* and *C*; see *SI Appendix*, Fig. S1 for schematic representations of these planes). For monkey S, the recording chamber was positioned just above the calcarine sulcus (dashed lines, Fig. 1*C*) to maximize the surface and depth of the primary visual cortex (V1) imaged (44–46) (Fig. 1*C*). For monkey T, the recording chamber was positioned above the lunate sulcus to image the contiguous visual areas V1, V2, and V3 in the same plane (45, 47, 48) (Fig. 1*C*). The fUS image was 10 mm in depth and 14 mm in width. On these anatomical fUS acquisitions, we can distinguish cerebral blood fluctuations within the blood vessels that appear in white in these images. This image enabled us to distinguish the anatomical sulci (calcarine sulcus and lunate sulcus) due to the presence of blood vessels as well as the deep microvascularization through the different cortical layers (white stripes).

We first examined whether CBV variations were modulated by the presentation of visual stimuli. CBV responses were measured in response to three different stimulus conditions: 1) when visual stimuli were presented for a short period (0.5 s), 2) for a longer period (2 s), and 3) when no visual stimuli were presented during the fixation period (*SI Appendix*, Fig. S2). For this first test, the visual stimulus was presented at the upper left of the visual field (a circle of 2 degrees of visual angle [DVA] diameter presented at azimuth −1 DVA and elevation −10 DVA). After just a single trial, the CBV increased significantly only within the posterior bank of the calcarine sulcus (Fig. 1*D*). The magnitude and localizations of these responses were similar for 0.5- and 2-s stimulations. No such CBV variation was observed when no visual stimulus was presented during the fixation period lasting for 2 s (*SI Appendix*, Fig. S2).

We then analyzed two important parameters for the imaging studies: the influences of the stimulation duration on the recorded hemodynamic responses, and the number of trials needed to obtain a clear activation map. We plotted the mean CBV signal within the responsive region of interest (ROI) (white dotted rectangles in activation maps; size, 50 × 18 pixels) according to the period of stimulation (blue, 0.5 s; red, 2 s). When only 10 trials were averaged (Fig. 1*D*, black rectangle), the CBV peak occurred after 2.5 s of the stimulation onset for both 0.5 and 2 s of stimulation duration. Hence, the mean CBV variation was averaged during 1 s centered on 2.5 s (Fig. 1*D*, gray rectangles) after the visual stimulus onset to compute all of the activation maps in this study. The amplitudes of average responses (for *n* = 10 trials) were similar and reached 16.27 ± 2.81% of CBV increase for a 0.5-s stimulation and 18.5 ± 4.65% for a 2-s stimulation. Moreover, the signal-to-noise ratios (SNRs) of the activation maps (Fig. 1*D*) were similar for the short (0.5 s) and long stimulations (2 s) (2.78 and 3.15 dB, respectively), so we decided to use the shortest stimulation duration (0.5 s) for the rest of the study.

The activation map was created from a defined number of trials to produce the clearest image of the responsive areas. After only a single 0.5-s trial, the responsive region was clearly detectable on the activation map with a response peak reaching 25.3%, although the map looked quite noisy (Fig. 1*D*). To investigate the influence of averaging on the response reliability, we generated combinations of activation maps computed with a different number of trials (1 to 20), comparing each of them with an activation map computed with 20 other trials. The comparison was made by counting each correctly classified pixel, namely all true positive and true negative pixels. After only 10 averaged repetitions, we reached 89.0% of correct classifications for monkey S across 24 sessions and 91.8% for monkey T across 32 sessions (Fig. 1*E*). In summary, we found that we could obtain accurate activation maps after only 10 trials with the 0.5-s visual stimulus. All subsequent measurements were therefore acquired with this shorter stimulation for at least 10 repeated trials.

### Retinotopic Mapping in the Whole Cortical Depth.

**Fig. 2.**
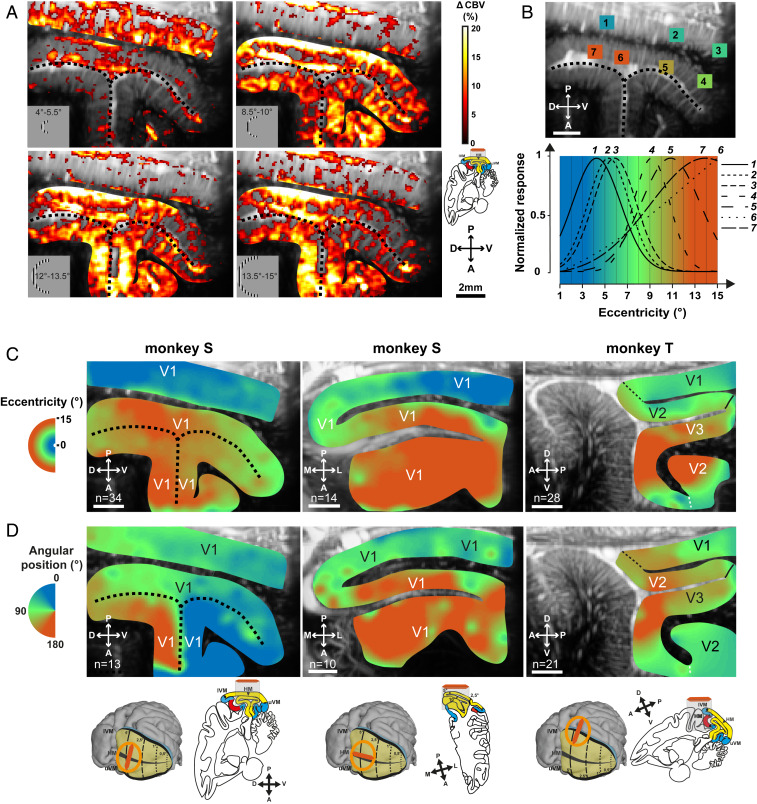
Retinotopic maps of the visual cortex with fUS imaging. (*A*) Activation maps obtained for four different stimulation eccentricities (from 4 to 15°) in V1 of monkey S (*n* = 34 averaged trials). Black dotted lines, calcarine sulcus. (*B*) Evolution of the normalized CBV responses for the seven chosen ROIs (1 to 7) as a function of the stimulus eccentricity indicated by color. Black dotted lines, calcarine sulcus. (*C* and *D*) Eccentricity (*C*) and angular (*D*) retinotopic maps reconstructed from activation maps as in *A*, obtained in a single session for each retinotopic map in the sagittal and transverse planes from monkey S (*Left* and *Middle*) and monkey T (*Right*); 34, 14, and 28 trials were averaged to compute eccentricity maps (*Left* to *Right*, respectively), and 13, 10, and 21 trials were averaged to compute angular maps (*Left* to *Right*, respectively). V1/V2 and V2/V3 borders were determined by atlas positions. The sagittal slices for both monkeys are the ones presented in Fig. 1*C*. (Scale bars, 2 mm.)

Having found such reliability over very few trials, we tested whether fUS imaging would be accurate enough to functionally map the cortex in awake monkeys within a single acquisition session. The retinotopic organization of the primate visual cortex is classically defined by mapping its visual sensitivity to a specific eccentricity and angular position in the visual field (49). We therefore tested whether presenting the stimulation locus in different positions of the visual field would shift the CBV variations across the visual cortex. The CBV variations were first measured in response to nine different visual stimulation eccentricities restricted to a half-ring in the left visual field. The range of the maximal cortical CBV responses was between 10 and 40%, as expected (*SI Appendix*, Fig. S3*A*). Fig. 2*A* presents four different activation maps when visual stimuli were presented, respectively, in the parafoveal position (*Top Left*, 4 to 5.5 DVA), at mideccentricities (*Top Right*, 8.5 to 10 DVA; *Bottom Left*, 11.5 to 13 DVA), and at a larger eccentricity (*Bottom Right*, 13 to 15 DVA). Despite some salt-and-pepper noise, all these CBV activation maps were different for the different visual field locations. To quantify these differences, we selected seven different ROIs (ROI size, 10 × 10 pixels) in this example along the activation maps (Fig. 2*B*). When the visual stimulation was located in the parafoveal region (4 to 5.5 DVA), CBV increased significantly for ROIs 1 to 3 compared with CBV responses in ROIs 4 to 7 that increased for more eccentric stimulations. This eccentricity selectivity is generalized for all pixels in *SI Appendix*, Fig. S3*B*. This map represents the eccentricities inducing the highest measured CBV responses. This observation is consistent with previous studies showing that superficial V1 processes visual information for low eccentricities, unlike deep V1 (11, 50). Moreover, some regions seemed less selective with a large eccentricity bandwidth (ROI 6, for example) compared with others with a sharp bandwidth (ROI 4, for example). This selectivity disparity is highlighted in *SI Appendix*, Fig. S3*C* representing the bandwidth of the Gaussian fitting for each pixel.

Fig. 2 *C *and *D* illustrates all cortical retinotopic maps for the two monkeys. These retinotopic maps were established with only one recording session for each map. For instance, the first eccentric map was generated with 34 correct trials per condition, resulting in an acquisition session lasting shorter than 1 h (Fig. 2*C*, *Left*). On this map, we observed a representation of the visual field with larger eccentricities represented through the calcarine sulcus in depth and the smaller eccentricities within superficial V1 (Fig. 2*C*, *Left*). The observation that eccentricity preference was largely unchanged within superficial V1 and within deep V1 in the sagittal imaging plane was expected, considering the organization of the visual cortex (Fig. 2*C*, *Left* and *Right*) (50). Moreover, the map of the transverse plane confirms the progressive evolution of the eccentricity projection of V1 within the medial fold (Fig. 2*C*, *Middle*) with a progression toward the foveal representation on the operculum (below 8 DVA; blue color) whereas deep V1 was more activated by more peripheral locations (over 8 DVA; orange color).

On the angular retinotopic maps, the sagittal plane view in monkey S (Fig. 2*D*, *Left*) illustrates that visual stimuli located in the upper visual field induce significant CBV variations in the ventral bank of V1 calcarine sulcus (represented in blue; angular position between 0 and 45°). By contrast, visual stimuli presented in the lower visual field induce CBV changes in the dorsal bank of V1 calcarine sulcus (represented in orange; angular position between 135 and 180°). No evolution of the angular retinotopic map within superficial V1 in the transverse plane was expected, considering the functional organization of the visual cortex (Fig. 2 *D*, *Middle*). In monkey T, the V1/V2 and V2/V3 borders can be localized with anatomical landmarks (51) (Fig. 2, V2 atlas delimitations). Previous studies have defined these borders as the respective vertical and horizontal meridian projections (Fig. 2*D*, *Right*, represented respectively in orange and green; the polar angle is centered on 180 and 90°) (52). However, on our retinotopic maps (Fig. 2*D*, *Right* and *SI Appendix*, Fig. S3 *C* and *D*), the representation of the lower vertical meridian, which is supposed to include the V1/V2 border, is not located at the known V1/V2 anatomical border as expected but appears deeper in the lunate sulcus. The responsive area to the vertical meridian does not delimit a precise position but rather a border confidence zone (*SI Appendix*, Fig. S4*B*, between the two arrows). This discrepancy with published anatomical data and our difficulty in locating this V1/V2 border can result from uncertainties of fixation limitations of fUS measurements (*Discussion*). Therefore, such functional studies provide an alternative means to examine the delimitations of cortical structures in living animals.

As indicated above, all these retinotopic maps were established after only one acquisition (about 1 h of experimental time). To illustrate the repeatability of the measurements, *SI Appendix*, Fig. S3 *E* and *F* presents two retinotopic maps generated in two different sessions on 2 different days. Note that the functional and anatomical maps were not fully identical, likely because the probe could not be secured in precisely the same position each day. Despite this problem, 77.8 and 89.1% of the pixels had the same eccentric preference value (±2 DVA) for, respectively, *SI Appendix*, Fig. S3 *E* and *F*, showing that across different behavioral sessions, we could obtain fairly similar retinotopic maps. Despite these variabilities across sessions and discrepancies with anatomical maps, these results are consistent with the conclusion that functional ultrasound imaging could be used to broadly map visual responses within different visual areas, even deep within the visual cortex.

### fUS Reveals Cortical Layer Selectivity of the Visual Cortex in Ocular Dominance Mapping.

**Fig. 3.**
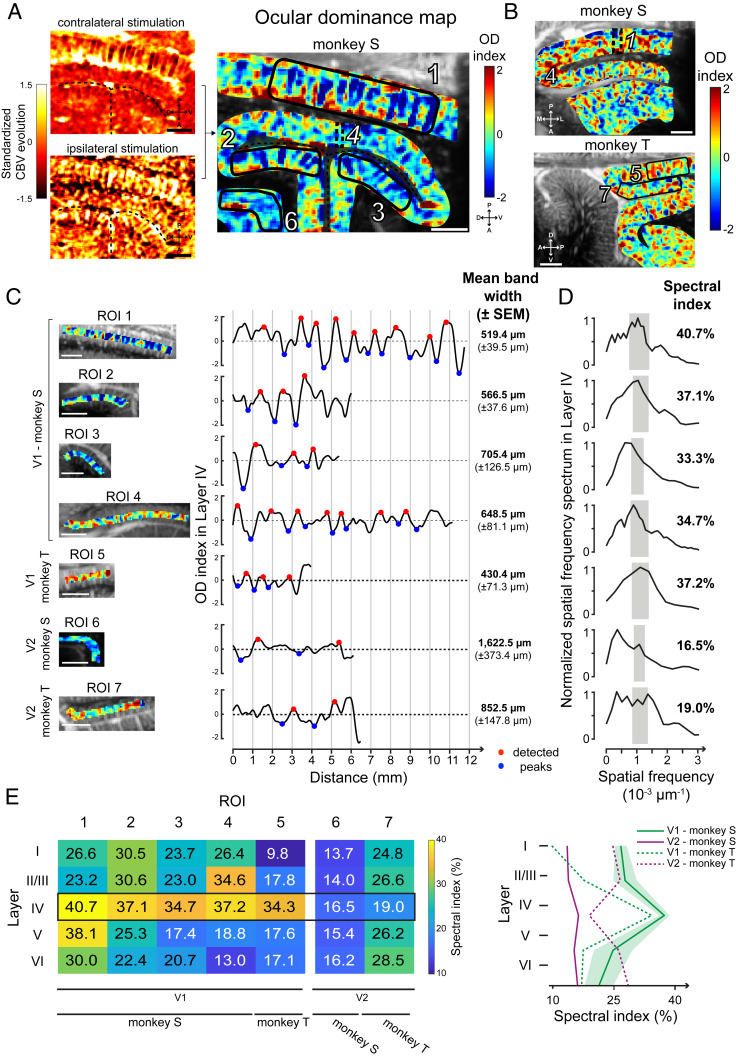
Ocular dominance maps in the visual cortex. (*A*) OD map (*Right*) obtained from the standardized CBV evolution in V1 for contralateral (*Upper*) and ipsilateral (*Lower*) stimulations in monkey S (*Left*). Black dotted lines, calcarine sulcus. This is the same sagittal slice as in Figs. 1*C* and 2. (*B*) OD maps obtained for monkey S in a transversal imaging plane and for monkey T in the sagittal plane (same slices as in Figs. 1*C* and 2). Black dotted lines, V1/V2 border (determined by an atlas). (*C*) Mean OD index across layer IV for each ROI (*Left*, black polygons in *A* and *B*) showing peaks (red and blue circles) used to compute the mean bandwidth. (*D*) Spatial frequency spectrum of layer IV for each ROI. The spectral index is computed integrating the spectrum in a bandwidth corresponding to a 350- to 700-µm OD bandwidth range (gray rectangles). (*E*) Table and curves with the spectral index computed and averaged for all layers and ROIs (shaded bars, SEM). (Scale bars, 2 mm.)

To further assess the spatial functional resolution of fUS imaging, we examined whether ocular dominance (OD) columns can be resolved with this new imaging technology. In macaque visual cortex, OD columns are typically described as the alternation of 400- to 700-µm-thick cortical bands preferring the input from one eye or the other (53–55). So we measured CBV variations during the presentation of a visual stimulus in the central visual field (limited to 15 DVA) when the ipsilateral eye was masked (Fig. 3*A*, *Top Left*), and we reproduced this acquisition masking only the contralateral eye (Fig. 3*A*, *Bottom Left*). A map revealing patterns similar to ocular dominance columns (Fig. 3 *A*, *Right*) was obtained by subtracting ipsilateral to contralateral evoked maps. We observed the presence of alternative complementary columns within V1 in both animals. Indeed, the pattern was present in superficial V1 (ROI 1) but also through the calcarine sulcus (ROIs 2 and 3). In contrast, we did not observe such columns at the roof of the calcarine sulcus. As visualization of ocular dominance columns is expected if the imaging plane is oriented perpendicular to OD bands, we performed observations in the same area but in the transverse plane (Fig. 3*B*, *Top*). In this transverse plane, the alternations became apparent at the roof of the calcarine sulcus (ROI 4), but they disappeared in the superficial V1 or in deep V1 (ROIs 1 to 3). The presence of this pattern similar to ocular dominance columns was confirmed in monkey T (Fig. 3*B*, *Bottom*) along the superficial V1 (ROI 5) even if they are less distinct than those in monkey S. Despite a patchy noisy pattern in V2, the V1/V2 difference on monkey T could be distinguished by the presence of columnar patterns in V1 (*SI Appendix*, Fig. S4*A*, black dotted line) while such a regular pattern cannot be defined in V2 for both monkeys (ROI 6 for monkey S and ROI 7 for monkey T). The reliability of these columnar patterns was tested by computing the map after shuffling ipsilateral with contralateral blocks (*SI Appendix*, Fig. S5*A*). In this analysis, the disappearance of the patterns confirmed that they are not artifacts due to one acquisition. Both activation maps (ipsilateral and contralateral) are clearly complementary in response, suggesting that these functional maps are robust. Moreover, without the manual cropping (*SI Appendix*, Fig. S5 *A*, *Left*), we can observe that the vertical columnar pattern is only visible within V1.

We then investigated whether the columnar patterns resulting from the cross-section we observed were similar to OD bands as previously described in the literature (53, 55, 56). To estimate the widths, we selected seven different ROIs in superficial V1 (1 and 5), deep V1 (2 to 4), and V2 (control, 6 and 7). For these ROIs, the OD index (computed by subtracting the CBV ipsilateral map from the CBV contralateral map; *Methods*) was plotted along layer IV, after segmenting V1 visual cortex according to Hässler’s scheme [Fig. 3*C*; see *SI Appendix*, Fig. S5*B* for layer segmentation (57)]. Periodic OD index oscillations were clearly visible for ROI 1 and the mean OD bandwidth in superficial V1 was found to be 519.4 µm (±39.5 µm). This band’s width was similar within the dorsal and ventral banks (ROI 2, 566.5 ± 37.6 µm; ROI 3, 705.4 ± 126.5 µm), with those of the roof of the calcarine sulcus (ROI 4, 648.5 ± 81.1 µm), and with those of monkey T’s superficial V1 (ROI 5, 430.4 ± 71.3 µm). These measurements accord well with the range of 400 to 700 µm classically reported for ocular dominance bandwidth (53–55). The OD index along V2 did not show the same range of OD bandwidth (ROI 6, 1622.5 ± 373.4 µm; ROI 7, 852.5 ± 147.8 µm). Spatial frequency spectra of the OD index in layer IV further confirmed the presence of a periodicity corresponding to the classical OD bandwidth for V1 ROIs (1 to 5) (Fig. 3*D*). Indeed, a peak was clearly visible around the spatial frequencies (SFs) corresponding to the classical 350- to 700-µm range of OD bandwidth for these V1 ROIs (represented by the gray rectangles in Fig. 3*D*: 7.1429 × 10^−4^ µm^−1^ < SF < 1.4 × 10^−3^ µm^−1^). By contrast, for V2, the selectivity peak was present at a very low spatial frequency in ROI 6 (SF = 1.631 × 10^−4^ µm^−1^) and the spectrum covered a large selectivity in spatial frequencies for ROI 7 (0 µm^−1^ < SF < 1.386 × 10^−3^ µm^−1^). To quantify these spectra within the theoretical OD bandwidth, we computed the “spectral index” as the proportion of the spectrum covering the classical 350- to 700-µm range of OD bandwidth (Fig. 3*D*, gray rectangles). This spectral index was superior to 30% for all V1 ROIs when it was lower than 19% for all V2 ROIs. This result was consistent with the notion that the classic OD bandwidths are only present in V1, suggesting that our observed columnar patterns could represent cross-sections of OD bands.

The precise spatial resolution of this imaging technique allows us to study the cortical layer segmentation. OD bands were mainly reported in cortical layer IVc (related to Brodmann’s scheme) in visual area V1 and interpreted as a layer specificity of thalamic projections (54, 55, 58). To further assess this layer selectivity, we computed the normalized spatial spectrum for all layers of each ROI to extract all spectral indexes (Fig. 3*E*). For V1 ROIs (1 to 5), this analysis revealed clear increases of the spectral indexes in layer IV compared with layers I, II/III, V, and VI. These peaks in layer IV were similar for both monkeys in V1 whereas there was no such an increase through layers in V2 (Fig. 3*E*, *Right*). These observations are in accordance with the first observations of Hubel and Wiesel (54, 59). This conclusion further supports that the observed columnar pattern represents a cross-section of OD bands. However, even if the spectral indexes were always in the highest range for layer IV (34.3 to 40.7%), some ROIs also showed high values within layers II/III (ROIs 2 and 4, respectively, 30.6 and 34.6%) and within layer V (ROI 1, 38.1%). This study suggests that OD bands could extend to layers II/III and V of the V1 visual cortex.

## Discussion

A key aim of this study was to test whether we could record and localize neuronal activity within the visual cortex of awake nonhuman primates at mesoscale (ranging from 100 μm to 2 cm) using the innovative imaging technique of functional ultrafast ultrasound imaging. Using different mapping protocols, we have shown that it is possible to generate retinotopic maps consistent with validated knowledge of the primary visual cortex of primates. This range of spatial resolution and the field of view in depth enabled us to distinguish columnar patterns highly reminiscent of ocular dominance selectivity of cortical layers throughout the primary visual cortex. This imaging technique surpasses some of the disadvantages of conventional techniques, such as fMRI and optical imaging methods, as we detail below.

### Deep Functional Mapping through All Cortical Layers of the Visual System.

The OD mapping obtained with fUS imaging demonstrates the clear advantage of an in vivo imaging modality with high spatiotemporal resolution at depth. Since Hubel and Wiesel first demonstrated the existence of OD cells in V1 with electrophysiological recordings (59), most subsequent studies have used autoradiographic methods which required that animals be killed (52, 55, 56, 58). In functional imaging, OD bands were also detected by optical imaging methods such as intrinsic-signal optical imaging (60), VSDI (61), or calcium imaging (23) but this demonstration was always limited to the superficial cortical layers because such optical methods cannot reach deep layers or the deep cortex. Although further studies are required to further confirm that the observed fUS columnar pattern corresponds to a cross-section of OD bands, we have shown that fUS imaging will likely surpass the limitation of optical techniques by mapping this columnar pattern in vivo to the depth of the entire cortex.

OD columns were classically described in layer IV of V1 (55) as confirmed by the recording of cells responding to the stimulation of one eye (59). Our observation of an extension of OD columns in layers II/III and V is consistent with previous anatomical studies showing some continuities between OD columns in layer IV and such OD columns in layer V or cytochrome oxidase (CO) blobs in layer II/III as indicated by autoradiography or immediate-early gene immunolabeling (52, 58, 62). However, in the visual cortex, the large blood vessels extend radially. As a consequence, one may wonder whether a vertical alignment of large blood vessels in V1 could artificially extend the columns in layers II/III and V. Indeed, blood circulating in a closed system generates a peripheral influence on the measured signal (63, 64). Although it is not possible to completely refute this hypothesis, it is not probable here. Our analysis of the ultrafast ultrasound technology filtered the signal produced by arterioles, capillaries, and venules based on their blood velocity (between 1 and 25 mm/s) and discriminated it from the influence of larger vertical vessels (65). The OD selectivity in layer IV cannot be explained by an SNR variation because the parameter which influences the fUS imaging more is the imaging depth (32) and, in this study, the ROIs were selected with different imaging depths and orientations. In agreement with previous anatomical studies, our observation of the columnar patterns provides in vivo functional evidence of the extension of OD bands from layers II/III to V.

The weak distinction of the columnar patterns in the deep cortex of V1 could be due to smaller column sizes below the resolution limit of the technology. OD bands are thinner for high eccentricities (53, 66, 67). OD bands parallel to the ultrasound probe could also provide an alternative explanation. This seems likely to be the case for the OD columns at the roof of the calcarine sulcus, as indicated by a previous study (55). Designing a new probe with a higher US frequency (30 MHz) would increase the spatial resolution for such measurements by a factor of 2 (68). Such an increase in spatial resolution could enable investigators to identify other functional structures such as the orientation selectivity columns in V1 or CO blobs, although the maximal imaging depth would be reduced (23, 61, 69–71).

### fUS Imaging Allows Fast and Easy Mapping.

In our study, we showed that 10 short stimulations (0.5 s) are sufficient to map cortical activity. This result represents a major advance on even the latest laminar fMRI techniques, which are less sensitive and more time-consuming using ultra-high-field MRI to study the visual cortex in humans (72, 73). Mapping the anatomic microvascularization with this ultrasound system is very fast: A single second is enough to capture one imaging plane with a spatial resolution of 110 × 100 × 400 μm^3^ and a field of view of 1.4 × 2 cm^2^ compared with a 7-T MRI which typically requires a couple of minutes to provide a 0.5-mm isotropic acquisition of the brain (74). A lower spatial resolution (0.7-mm isotropic) was described in another human V1 study using 7-T fMRI coupled to two high-density 16-channel surface coils for a similar field of view (1.31 × 1.20 cm^2^) (75). Moreover, MRI acquisitions can be stressful for the monkey and there are different electromagnetic constraints (5–7), whereas fUS imaging can be easily performed on an awake behaving monkey with no more constraints than typical electrophysiological approaches. Finally, Boido et al. have recently showed a high increase of sensitivity with fUS imaging compared with fMRI (17.2 T) in the same animals (76). Therefore, fUS imaging can thus easily be exploited for anatomical acquisitions in recording chambers to guide injections or electrode implantations, especially at depth.

Yet, if a stereotaxic localization of functional structures revealed by fUS acquisitions is needed, an anatomical MRI should be considered to perfectly determine the position and the orientation of the fUS imaging plane within the brain. In the absence of an MRI image, our retinotopic maps have to be considered with caution concerning the absolute values. Moreover, the protocols were not designed to map the retinotopic representation with accuracy. Indeed, the tolerance window for the fixation task was slightly too large (up to 2.8 DVA in diameter, causing, respectively, a mean of 0.40 and 0.43 DVA SD in *x* and *y* axes for the eight retinotopic sessions) and the sizes of the stimuli were too broad (respectively, 2 DVA and 15° for eccentricity and angular mapping). Although retinotopic maps are consistent with previous studies, this could explain that some observations are not fully in agreement with classic anatomical maps as for the V1/V2 border (*SI Appendix*, Fig. S4 *B*–*D*). However, the observed columnar patterns suggested a better localization of the V1/V2 border given OD alternations are not visible within the deep lunate sulcus (*SI Appendix*, Fig. S4*A*).

Some functional structures are anatomically oriented but fUS only records one 400-μm-thick imaging plane oriented in depth. Consequently, it would be tricky to image functional structures when their orientation—or position—is parallel to the imaging plane. This spatial restriction could be compared with the deep-axis limitation with optical imaging. The solution is, when possible, to perpendicularly rotate or shift the probe within the recording chamber as we did to reveal the OD columns at the roof of the calcarine sulcus for monkey S, or to image the cortex at a different angle. A 3D reconstruction would then be possible, as previously achieved with parallel imaging planes (4, 29, 65).

Given the good SNR of this method, fUS imaging could quickly become the gold standard for mesoscopic functional mapping of a region prior to further electrophysiological studies, deep-brain stimulation implantation, or localized drug delivery. Electrophysiological methods are too time-consuming for such preliminary mapping (44, 67, 77–79), while fUS imaging can generate a retinotopic map with only a 1-h session. In a perfect session, with only correct fixations for all trials, only 420 s of acquisition would be needed to reconstruct the polar angle map with fUS imaging, compared with the 3,200 s required using fMRI (more than seven times longer) (80). The superior spatial resolution of fUS imaging has enabled mapping a columnar pattern in nonhuman primates, likely a cross-section of OD bands, an achievement currently unattainable with fMRI.

### The Significance of the CBV Signal Measured with fUS Imaging.

By definition, fUS signal is the power Doppler signal extracted from the acquisition sequence as described in *Methods*. It has been demonstrated that the power Doppler signal is proportional to the number of moving red blood cells in a sample volume (roughly a 100 × 100 × 400-µm voxel in this study) (81). Considering that some assumptions are respected here such as a constant hematocrit, constant scattering properties of blood, and a good clutter filtering (33), fUS signal is therefore proportional to the CBV (82). In this study, the larger blood vessel participation was mostly suppressed according to the Doppler frequency shift and therefore on axial velocity of red blood cells as described by Macé and colleagues (82). However, no cerebral blood flow (CBF) estimation is used in the fUS signal processing. Given that CBV and power Doppler signal are based on approximations (82), this could result in some other flow parameters, such as CBF, to be mixed in the power Doppler signal. Yet, it seems rather unlikely that they would be predominant over CBV.

Moreover, Rungta and colleagues recently measured the red blood cell velocity variations across the different vascular compartments (from the juxtasynaptic capillary backward to the feeding pial arteriole) in the mouse olfactory bulb, in response to odor (83). Despite heterogeneous blood velocity increases according to vessel type, the largest proportional increase of this hemodynamic parameter was observed in the juxtasynaptic capillaries (83), providing evidence for the very high sensitivity of measuring CBV evolution to accurately localize neural activation. Furthermore, with the dynamic filtering out of large vessels in fUS imaging, their smaller changes in blood velocities may explain why no spatial blurring of the functional response was reported in recent studies distinguishing small cortical areas with fUS imaging (4, 34, 41). For instance, Bimbard and colleagues were able to discriminate pixels spaced by 300 µm with their tuning curves while characterizing the tonotopic map in the awake ferret with fUS imaging (41). Such a resolution is compatible with layer and OD column discrimination in the visual cortex of primates.

Further studies are needed to clearly evaluate the contribution of the different vascular structures to CBV signal. Indeed, noise can be seen on single measures but averaging already greatly decreases this noise level (Fig. 1 and *SI Appendix*, Fig. S2). This noise was mainly observed in superficial and deep laminar locations within the cortex. Comparing fUS imaging with other modalities such as electrophysiology or optical imaging techniques would help to better understand the significance of the CBV signal measured by fUS imaging (76).

Other features could affect the quality of fUS signal. The main cause of expected artifacts with fUS imaging is related to movement of tissues given that CBV is extracted from the power Doppler signal. By contrast to MRI, image registration cannot solve this problem in fUS acquisitions but such movement artifact is easily detected and removed. Even if movements linked to breathing and heartbeats are mostly filtered, there are still macroscopic and irregular movements provoked by the monkeys which are spread to the brain tissues. The interfaces (dura/cortex, cortex/cortex) seem to generate more SNR variability and this could be explained by the fact that these interfaces facilitate sliding between tissues (84). More generally, the SNR variability will depend partly on the biomechanical characteristic differences of tissues within the imaging plane (85).

### A Slightly Invasive and Long-Term Imaging Technique.

Although this fUS imaging technique requires a craniotomy for primate studies—though this is not necessary for rodents (38, 86)—the dura is kept intact. Ultrasonic waves propagate without major reflections or diffraction effects through soft tissues. This property means that dura, coagulated blood, or other optical barriers do not significantly affect the SNR. Over a period of several months, we observed a slight decrease of the SNR, which may be due to dura thickening but remained negligible for our purposes. Therefore, fUS imaging provides a powerful imaging technique for long-term sequential studies. Its field of application is adapted to the mapping of superficial and deep cortical areas (up to 2 cm deep). Increasing the depth of acquisition as far as subcortical areas such as the thalamus would require reducing the US frequency, which would decrease the spatial resolution. With a 6-Mhz-frequency probe, we were able to image throughout the macaque brain (40).

### A Continuum of Brain Imaging Technology.

Functional brain imaging is becoming a major field of technological innovation to investigate brain function in normal and pathological conditions. The resolution of fMRI is increasing further by increasing the power of the magnetic field. However, such equipment is difficult to introduce into surgical rooms and laboratories. Recently, magnetoencephalography was adapted to generate measurements in freely moving patients with a very high temporal resolution (7.7 ms) but a low spatial resolution (centimeters) (87). By contrast, we show here that fUS imaging provides a very good spatial resolution at the mesoscale level through cortical depth as long as a skull window can be achieved. This new technology should therefore fill a major gap in brain mapping within surgical rooms and laboratories when optical techniques are not relevant due to depth concerns. Although such usage remains limited, fUS imaging technology has already been demonstrated on human babies during transfontanellar imaging (42) or on human patients for perioperative imaging during brain surgery (43).

## Methods

### Experimental Apparatus.

We collected data from two rhesus primates (*Macaca mulatta*; “monkey S,” a male aged 13 y, and “monkey T,” a female aged 11 y), weighing 13 and 9 kg, respectively. These monkeys were individually housed and handled in strict accordance with the recommendations of the Weatherall Report on good animal practice. All experiments were conducted after validation of the European Council Directive (2010/63/EU) and the study was approved by the French ministry and the institutional and regional committees for animal care (Committee C. Darwin, registration no. 9013).

Titanium head posts and recording chambers were implanted under aseptic conditions as described by Valero-Cabre and colleagues (88). Briefly, following preoperative analgesia with butorphanol (0.2 mg/kg, intramuscular; i.m.), anesthesia was induced with ketamine (0.3 mg/kg) and dexmedetomidine (0.015 mg/kg, i.m.) and maintained with isoflurane (1 to 2%). The animals were mechanically ventilated (Hallowell EMC; model 2000). Heart rate, temperature, respiration, and peripheral oxygen saturation (SpO2) were monitored throughout. Buprenorphine (0.015 mg/kg, i.m.) was routinely given at the end of the surgery (to reverse butorphanol effects), together with prophylactic long-acting i.m. amoxicillin 15 mg/kg (Clamoxyl LA) or oxytetracycline 20 mg/kg (Duphacycline LA). The head post was positioned in the medial line and was sufficiently rostral to allow the subsequent implantation of a recording chamber above the primary visual area. A postoperative recovery period of 6 wk was observed before fixating the animal’s head.

After the recovery period, the animals were trained to perform a passive fixation task (Fig. 1*A*). When their performance reached a significant threshold, a recording chamber was implanted above the visual cortex (Fig. 1*B*) based on MRI scans (3-T MRI; T2* sequence; 0.5-mm isotropic). The monkeys were deeply anesthetized and monitored during surgery. The recording chamber (Crist Instruments; CILUX chamber 6-IAM-J0) was positioned and centered above the right calcarine sulcus for monkey S (medial-lateral [ML] +7 mm; anterior-posterior [AP] −22 mm) and above the right lunate sulcus for monkey T (ML +7 mm; AP −10 mm; dorsal-ventral [DV] +38 mm). The dura mater was kept intact during the procedures.

### Visual Stimuli.

Each behavioral session lasted a maximum of 2 h. The animals were seated in a primate chair (Crist Instruments) with their head fixed and placed in front of a computer screen (Iiyama; ProLite XB2783HSU; gamma-corrected, resolution 1,920 × 1,080 pixels, running at 60 Hz) 53 cm away in a darkened booth. Mean screen luminance was controlled (9 cd/m^2^). Eye position was monitored using an EyeLink 1000 infrared eye-tracking system (SR Research). Experiments were controlled by EventIDE software (OkazoLab). The monkey started the trial by fixating a central green square subtending 0.2 degrees of visual angle for 500 ms within a tolerance window of 1 to 1.4 DVA. A visual stimulus of the peripheral location was then presented for 0.5 or 2 s. We used drifting sinusoidal gratings as visual stimuli with a fixed temporal frequency (three cycles per second) and a fixed spatial frequency (one cycle per degree). The animals were rewarded by a small drop of liquid (water) at the end of each correct fixation trial. We imposed an intertrial interval of 3 s. We also used control trials with the same temporal organization but without any peripheral visual stimulus (*SI Appendix*, Fig. S2). All conditions (visual and control) were randomly interleaved. We collected an average of 20 trials per visual condition.

We obstructed the right eye of both monkeys with an opaque visor to generate the retinotopic maps of the visual cortex. We focused on recording three different functional maps: one based on the eccentricity, one based on the angular positions, and one for the ocular dominance columns. For the eccentricity map, we used nine different visual conditions with eccentricities ranging from 1.5 to 15 DVA. Each stimulus was defined as a hemiconcentric band of 1.5° width centered on the central fixation point. For the angular position maps, we used 12 different visual stimuli: Each stimulus was defined by 15° of angular width, in the left visual field, from 1.5 to 15 DVA of eccentricity. To reveal the ocular dominance columns within the primary visual cortex, either the right or left eye of the monkey was masked and we used a “full-field” vertical grating (spatial frequency, one cycle per degree; temporal frequency, three cycles per second) from 1.5 to 15 DVA of eccentricity.

### fUS Acquisition Sequence.

We recorded fUS images using a linear ultrasound probe (custom design, 128 elements, 15 MHz, 110-μm pitch; Vermon) driven by a modified ultrafast ultrasound research scanner (256 electronic channels, 60-MHz sampling rate; Supersonic Imagine). Full details of the technical procedures and statistical analysis of our fUS imaging technique have already been described in several previous publications from our group (29, 30, 34). Briefly, fUS images were acquired by repeated emissions of a set of 15 planar ultrasonic waves (pulse-repetition frequency, 7,500 Hz) tilted with different angles ranging from −14 to 14° (89) in 2° steps in order to obtain one high-quality ultrasound image. To sample cerebral blood volume variations, we repeated this sequence 200 times at a 500-Hz frame rate, corresponding to a 400-ms acquisition time. In order to remove tissue-motion artifacts from the dataset, we applied a recently developed clutter-filter technique on rats based on singular-value decomposition (33) without any modifications for application to the awake monkey. Finally, one ultrasensitive Doppler image was formed for each ultrafast data block with a sampling rate of 1 Hz by averaging the 200 compounded and filtered ultrasonic images. Therefore, we extracted CBVs from ultrasensitive Doppler images with a sampling rate of one image per second. During a behavioral session of 60 min, we obtained 3,600 images (98 × 128 pixels). We could image 14 mm along the cortical surface and 10 mm in depth. Our spatial resolution was 0.11 × 0.1 mm and the width was 0.4 mm.

When working on retinotopic maps, we usually only performed all fUS imaging acquisitions required for a complete map during the behavioral session. For ocular dominance maps, we performed all required acquisitions on both eyes during the session.

### Data Processing.

We then analyzed all of the fUS image acquisitions using Matlab (version R2017b; MathWorks). CBV signals were 10 times extrapolated (cubic spline interpolation; temporal resolution, 0.1 s) and 3D smoothed (convolution kernel, [3 3 3]; box filter, SD 0.65). A log file created by EventIDE during each behavioral session was used to extract CBV signal with the beginning of each correct trial. This signal was then normalized with the baseline (the average of the signal 5 s before the onset of the visual stimulus). The peak CBV response was detected ∼2.5 s after the onset of the visual stimulus (Fig. 1*D*). The response of a pixel (0.11 × 0.1 × 0.4 mm^3^) was thus quantified as the mean CBV level within the 1-s period from 2 to 3 s after visual stimulus onset for the first experiment (Fig. 1), within the 0.5-s period from 2.5 to 3 s after visual stimulus onset for the retinotopic maps (Fig. 2), and within the 1-s period from 2.5 to 3.5 s after visual stimulus onset for the OD maps (Fig. 3). These temporal parameters were chosen empirically to better highlight the desired functional map.

To compute the proportion of correct classification (Fig. 1*E*), we used only sessions in which at least 20 repetitions of the same visual condition occurred (*n* = 32 for monkey T; *n* = 24 for monkey S). We split sessions with more than 40 repetitions to obtain notional sessions of 40 trials. We then randomly selected 20 trials within the session to compute a reference activation map, and used a threshold of a 10% increase in CBV to binarize the map. This threshold was defined based on empirical estimation and its robustness to compute all functional maps across the two animals and all of the sessions. Moreover, a 10% increase corresponds to 1 to 2 SDs of supplementary eye field activity in macaque as described in a previous study (40). For a fixed number of averaged trials (1 to 20), we selected randomly 100 trial combinations (excluding trials used to generate the reference activation map) to compute the different binary activation maps using the same threshold (10% ΔCBV). We then measured the mean proportion of correct classification summing only true positive and negative pixels through the whole map compared with the reference map. We repeated this process for each number of averaged trials (1 to 20) and for each session. We used the least-squares method to fit a Naka–Rushton model on our data.

To reconstruct retinotopic maps, a 3D data matrix was constructed with the different activation maps for each visual condition. A Gaussian fitting was applied for each pixel and the coefficient of determination was computed to the threshold (*R*^2^ > 0.02) of the relevant pixels (the colored pixels in *SI Appendix*, Fig. S3*D*). Only the pixels with retinotopically modulated responses (i.e., with Gaussian behavior) are relevant to the mapping of retinotopic organization. In order to increase readability of the images, after obtaining the peaking index map (index of the fitted Gaussian peak for each pixel; *SI Appendix*, Fig. S3*B*), a 2D median filter, an interpolation (to refill empty pixels), and a 2D mean filter (averaging with the 2 neighboring pixels) were applied. However, retinotopic organization was also clearly present before smoothing (*SI Appendix*, Fig. S3*B*). The map was then manually cropped to only show the responses in the cortex (*SI Appendix*, Fig. S3 *E*, *Left*).

To generate the OD map, we computed the two activation maps (ipsilateral and contralateral) and then standardized them. We then subtracted the ipsilateral map from the contralateral map to obtain the OD map with the OD index values. We manually cropped these maps to reveal only the visual cortex. The layer segmentation was performed by indicating manually the top and the bottom of the cortex of the region of interest, computing the distance for each pixel between both, and indexing them using Hässler’s scheme (57). The evolution of the OD index across the cortex represents the centered smoothing spline fitting (least-squares method) of the data within the considered layer. The distance corresponds to the distance from the first intracortical column to the far left of the ROI (the far anterior or lateral). The extrema (blue and red dots in Fig. 3*C*) were obtained with a function finding the local extrema with a 0.75-OD index minimal peak prominence and an absolute value superior to a 0.25-OD index. The mean bandwidths were computed using the averaged half-distance between two maxima and two minima. We then computed the spatial spectrum for each layer of each ROI with a fast Fourier transform. The spatial spectra were filtered with a moving average (with three samples) and normalized by their maximum. We defined the spectral index as the proportion of the spectrum covering the 350- to 700-µm bandwidth.

To check the robustness of the OD map, we shuffled the ipsilateral blocks with the contralateral blocks. To do this, we first averaged all even blocks (ipsilateral and contralateral) to obtain a first activation map. We then used all residual odd blocks (ipsilateral and contralateral) to compute the second activation map. We then applied the same algorithm as described above to compute this final artificial OD map from these two “even and odd block” maps.

We decided to compute the spectral index in OD maps by considering columns with a 350- to 700-µm width. Indeed, Horton and Hocking reported a mean column width along the V1 border around 536 ± 81.8 µm (based on six monkeys) (53). Here we chose to consider 2 SDs (so, an interval of about [370 to 700]) and, given they did not compute the OD bandwidth within the calcarine sulcus [where the mean OD bandwidth can be divided by a factor of about 2 (55)], we decided to slightly extend the interval for low values (350–700 µm).

### Data Availability.

Data are available from the Open Science Framework database (90). Associated protocols, codes, and materials discussed in the paper will be made available to readers upon reasonable request.

## Supplementary Material

Supplementary File

## References

[1] Felleman D. J., Van Essen D. C., Distributed hierarchical processing in the primate cerebral cortex. Cereb. Cortex 1, 1–47 (1991). 1822724 10.1093/cercor/1.1.1-a

[2] Callaway E. M., Luo L., Monosynaptic circuit tracing with glycoprotein-deleted rabies viruses. J. Neurosci. 35, 8979–8985 (2015). 26085623 10.1523/JNEUROSCI.0409-15.2015 PMC4469731

[3] Kerr J. N. D., Denk W., Imaging in vivo: Watching the brain in action. Nat. Rev. Neurosci. 9, 195–205 (2008). 18270513 10.1038/nrn2338

[4] Macé É.., Whole-brain functional ultrasound imaging reveals brain modules for visuomotor integration. Neuron 100, 1241–1251.e7 (2018). 30521779 10.1016/j.neuron.2018.11.031 PMC6292977

[5] Tsao D. Y., Moeller S., Freiwald W. A., Comparing face patch systems in macaques and humans. Proc. Natl. Acad. Sci. U.S.A. 105, 19514–19519 (2008). 19033466 10.1073/pnas.0809662105 PMC2614792

[6] Vanduffel W.., Visual motion processing investigated using contrast agent-enhanced fMRI in awake behaving monkeys. Neuron 32, 565–577 (2001). 11719199 10.1016/s0896-6273(01)00502-5

[7] Yue X., Nasr S., Devaney K. J., Holt D. J., Tootell R. B. H., fMRI analysis of contrast polarity in face-selective cortex in humans and monkeys. Neuroimage 76, 57–69 (2013). 23518007 10.1016/j.neuroimage.2013.02.068 PMC3647014

[8] Benson N. C.., The retinotopic organization of striate cortex is well predicted by surface topology. Curr. Biol. 22, 2081–2085 (2012). 23041195 10.1016/j.cub.2012.09.014 PMC3494819

[9] Engel S. A.., fMRI of human visual cortex. Nature 369, 525 (1994). 8031403 10.1038/369525a0

[10] Henriksson L., Karvonen J., Salminen-Vaparanta N., Railo H., Vanni S., Retinotopic maps, spatial tuning, and locations of human visual areas in surface coordinates characterized with multifocal and blocked fMRI designs. PLoS One 7, e36859 (2012). 22590626 10.1371/journal.pone.0036859 PMC3348898

[11] Blasdel G., Campbell D., Functional retinotopy of monkey visual cortex. J. Neurosci. 21, 8286–8301 (2001). 11588200 10.1523/JNEUROSCI.21-20-08286.2001 PMC6763878

[12] Heider B., Jandó G., Siegel R. M., Functional architecture of retinotopy in visual association cortex of behaving monkey. Cereb. Cortex 15, 460–478 (2005). 15749989 10.1093/cercor/bhh148 PMC1945172

[13] Ramsden B. M., Hung C. P., Roe A. W., Real and illusory contour processing in area V1 of the primate: A cortical balancing act. Cereb. Cortex 11, 648–665 (2001). 11415967 10.1093/cercor/11.7.648

[14] Ts’o D. Y., Frostig R. D., Lieke E. E., Grinvald A., Functional organization of primate visual cortex revealed by high resolution optical imaging. Science 249, 417–420 (1990). 2165630 10.1126/science.2165630

[15] Vanzetta I., Slovin H., Omer D. B., Grinvald A., Columnar resolution of blood volume and oximetry functional maps in the behaving monkey: Implications for fMRI. Neuron 42, 843–854 (2004). 15182722 10.1016/j.neuron.2004.04.004

[16] Chemla S.., Improving voltage-sensitive dye imaging: With a little help from computational approaches. Neurophotonics 4, 31215 (2017). 10.1117/1.NPh.4.3.031215 PMC5438098 28573154

[17] Chen Y., Geisler W. S., Seidemann E., Optimal decoding of correlated neural population responses in the primate visual cortex. Nat. Neurosci. 9, 1412–1420 (2006). 17057706 10.1038/nn1792 PMC1851689

[18] Grinvald A., Lieke E. E., Frostig R. D., Hildesheim R., Cortical point-spread function and long-range lateral interactions revealed by real-time optical imaging of macaque monkey primary visual cortex. J. Neurosci. 14, 2545–2568 (1994). 8182427 10.1523/JNEUROSCI.14-05-02545.1994 PMC6577512

[19] Meirovithz E.., Population response to contextual influences in the primary visual cortex. Cereb. Cortex 20, 1293–1304 (2010). 19759123 10.1093/cercor/bhp191

[20] Muller L., Reynaud A., Chavane F., Destexhe A., The stimulus-evoked population response in visual cortex of awake monkey is a propagating wave. Nat. Commun. 5, 3675 (2014). 24770473 10.1038/ncomms4675 PMC4015334

[21] Reynaud A., Masson G. S., Chavane F., Dynamics of local input normalization result from balanced short- and long-range intracortical interactions in area V1. J. Neurosci. 32, 12558–12569 (2012). 22956845 10.1523/JNEUROSCI.1618-12.2012 PMC6621242

[22] Murakami T., Yoshida T., Matsui T., Ohki K., Wide-field Ca(2+) imaging reveals visually evoked activity in the retrosplenial area. Front. Mol. Neurosci. 8, 20 (2015). 26106292 10.3389/fnmol.2015.00020 PMC4458613

[23] Nauhaus I., Nielsen K. J., Callaway E. M., Efficient receptive field tiling in primate V1. Neuron 91, 893–904 (2016). 27499086 10.1016/j.neuron.2016.07.015 PMC5384649

[24] Zhuang J.., An extended retinotopic map of mouse cortex. eLife 6, e18372 (2017). 28059700 10.7554/eLife.18372 PMC5218535

[25] Helmchen F., Denk W., Deep tissue two-photon microscopy. Nat. Methods 2, 932–940 (2005). 16299478 10.1038/nmeth818

[26] Oheim M., Beaurepaire E., Chaigneau E., Mertz J., Charpak S., Two-photon microscopy in brain tissue: Parameters influencing the imaging depth. J. Neurosci. Methods 111, 29–37 (2001). 11574117 10.1016/s0165-0270(01)00438-1

[27] Hinds O. P.., Accurate prediction of V1 location from cortical folds in a surface coordinate system. Neuroimage 39, 1585–1599 (2008). 18055222 10.1016/j.neuroimage.2007.10.033 PMC2258215

[28] Stensaas S. S., Eddington D. K., Dobelle W. H., The topography and variability of the primary visual cortex in man. J. Neurosurg. 40, 747–755 (1974). 4826600 10.3171/jns.1974.40.6.0747

[29] Gesnik M.., 3D functional ultrasound imaging of the cerebral visual system in rodents. Neuroimage 149, 267–274 (2017). 28167348 10.1016/j.neuroimage.2017.01.071 PMC5387157

[30] Macé E.., Functional ultrasound imaging of the brain. Nat. Methods 8, 662–664 (2011). 21725300 10.1038/nmeth.1641

[31] Rau R.., 3D functional ultrasound imaging of pigeons. Neuroimage 183, 469–477 (2018). 30118869 10.1016/j.neuroimage.2018.08.014

[32] Bercoff J.., Ultrafast compound Doppler imaging: Providing full blood flow characterization. IEEE Trans. Ultrason. Ferroelectr. Freq. Control 58, 134–147 (2011). 21244981 10.1109/TUFFC.2011.1780

[33] Demené C.., Spatiotemporal clutter filtering of ultrafast ultrasound data highly increases Doppler and fUltrasound sensitivity. IEEE Trans. Med. Imaging 34, 2271–2285 (2015). 25955583 10.1109/TMI.2015.2428634

[34] Osmanski B.-F., Pezet S., Ricobaraza A., Lenkei Z., Tanter M., Functional ultrasound imaging of intrinsic connectivity in the living rat brain with high spatiotemporal resolution. Nat. Commun. 5, 5023 (2014). 10.1038/ncomms6023 PMC4205893 25277668

[35] Osmanski B. F.., Functional ultrasound imaging reveals different odor-evoked patterns of vascular activity in the main olfactory bulb and the anterior piriform cortex. Neuroimage 95, 176–184 (2014). 24675645 10.1016/j.neuroimage.2014.03.054

[36] Urban A.., Chronic assessment of cerebral hemodynamics during rat forepaw electrical stimulation using functional ultrasound imaging. Neuroimage 101, 138–149 (2014). 25008960 10.1016/j.neuroimage.2014.06.063

[37] Sieu L.-A.., EEG and functional ultrasound imaging in mobile rats. Nat. Methods 12, 831–834 (2015). 26237228 10.1038/nmeth.3506 PMC4671306

[38] Tiran E.., Transcranial functional ultrasound imaging in freely moving awake mice and anesthetized young rats without contrast agent. Ultrasound Med. Biol. 43, 1679–1689 (2017). 28476311 10.1016/j.ultrasmedbio.2017.03.011 PMC5754333

[39] Urban A.., Real-time imaging of brain activity in freely moving rats using functional ultrasound. Nat. Methods 12, 873–878 (2015). 26192084 10.1038/nmeth.3482

[40] Dizeux A.., Functional ultrasound imaging of the brain reveals propagation of task-related brain activity in behaving primates. Nat. Commun. 10, 1400 (2019). 30923310 10.1038/s41467-019-09349-w PMC6438968

[41] Bimbard C.., Multi-scale mapping along the auditory hierarchy using high-resolution functional ultrasound in the awake ferret. eLife 7, e35028 (2018). 29952750 10.7554/eLife.35028 PMC6039176

[42] Demene C.., Functional ultrasound imaging of brain activity in human newborns. Sci. Transl. Med. 9, eaah6756 (2017). 29021168 10.1126/scitranslmed.aah6756

[43] Imbault M., Chauvet D., Gennisson J.-L., Capelle L., Tanter M., Intraoperative functional ultrasound imaging of human brain activity. Sci. Rep. 7, 7304 (2017). 28779069 10.1038/s41598-017-06474-8 PMC5544759

[44] Daniel P. M., Whitteridge D., The representation of the visual field on the cerebral cortex in monkeys. J. Physiol. 159, 203–221 (1961). 13883391 10.1113/jphysiol.1961.sp006803 PMC1359500

[45] Wandell B. A., Dumoulin S. O., Brewer A. A., Visual field maps in human cortex. Neuron 56, 366–383 (2007). 17964252 10.1016/j.neuron.2007.10.012

[46] Huff T., Mahabadi N., Tadi P., Neuroanatomy, Visual Cortex, (StatPearls Publishing LLC, Treasure Island, FL, 2020). 29494110

[47] Gattass R., Gross C. G., Sandell J. H., Visual topography of V2 in the macaque. J. Comp. Neurol. 201, 519–539 (1981). 7287933 10.1002/cne.902010405

[48] Zeki S. M., Uniformity and diversity of structure and function in rhesus monkey prestriate visual cortex. J. Physiol. 277, 273–290 (1978). 418176 10.1113/jphysiol.1978.sp012272 PMC1282389

[49] Tootell R. B.., Functional analysis of primary visual cortex (V1) in humans. Proc. Natl. Acad. Sci. U.S.A. 95, 811–817 (1998). 9448245 10.1073/pnas.95.3.811 PMC33802

[50] Tootell R. B., Silverman M. S., Switkes E., De Valois R. L., Deoxyglucose analysis of retinotopic organization in primate striate cortex. Science 218, 902–904 (1982). 7134981 10.1126/science.7134981

[51] Saleem K. S., Logothetis N. K., A Combined MRI and Histology Atlas of the Rhesus Monkey Brain in Stereotaxic Coordinates, (Academic Press, 2012).

[52] Tootell R. B. H., Hamilton S. L., Silverman M. S., Switkes E., Functional anatomy of macaque striate cortex. I. Ocular dominance, binocular interactions, and baseline conditions. J. Neurosci. 8, 1500–1530 (1988). 3367209 10.1523/JNEUROSCI.08-05-01500.1988 PMC6569205

[53] Horton J. C., Hocking D. R., Intrinsic variability of ocular dominance column periodicity in normal macaque monkeys. J. Neurosci. 16, 7228–7239 (1996). 8929431 10.1523/JNEUROSCI.16-22-07228.1996 PMC6578935

[54] Hubel D. H., Wiesel T. N., Laminar and columnar distribution of geniculo-cortical fibers in the macaque monkey. J. Comp. Neurol. 146, 421–450 (1972). 4117368 10.1002/cne.901460402

[55] LeVay S., Connolly M., Houde J., Van Essen D. C., The complete pattern of ocular dominance stripes in the striate cortex and visual field of the macaque monkey. J. Neurosci. 5, 486–501 (1985). 3973679 10.1523/JNEUROSCI.05-02-00486.1985 PMC6565187

[56] Wiesel T. N., Hubel D. H., Lam D. M. K., Autoradiographic demonstration of ocular-dominance columns in the monkey striate cortex by means of transneuronal transport. Brain Res. 79, 273–279 (1974). 4423575 10.1016/0006-8993(74)90416-8

[57] Balaram P., Kaas J. H., Towards a unified scheme of cortical lamination for primary visual cortex across primates: Insights from NeuN and VGLUT2 immunoreactivity. Front. Neuroanat. 8, 81 (2014). 25177277 10.3389/fnana.2014.00081 PMC4133926

[58] Kennedy C.., Metabolic mapping of the primary visual system of the monkey by means of the autoradiographic [^14^C]deoxyglucose technique. Proc. Natl. Acad. Sci. U.S.A. 73, 4230–4234 (1976). 825861 10.1073/pnas.73.11.4230 PMC431397

[59] Hubel D. H., Wiesel T. N., Receptive fields and functional architecture of monkey striate cortex. J. Physiol. 195, 215–243 (1968). 4966457 10.1113/jphysiol.1968.sp008455 PMC1557912

[60] Kaskan P. M., Lu H. D., Dillenburger B. C., Roe A. W., Kaas J. H., Intrinsic-signal optical imaging reveals cryptic ocular dominance columns in primary visual cortex of New World owl monkeys. Front. Neurosci. 1, 67–75 (2007). 18974855 10.3389/neuro.01/1.1.005.2007 PMC2518048

[61] Blasdel G. G., Salama G., Voltage-sensitive dyes reveal a modular organization in monkey striate cortex. Nature 321, 579–585 (1986). 3713842 10.1038/321579a0

[62] Takahata T., Higo N., Kaas J. H., Yamamori T., Expression of immediate-early genes reveals functional compartments within ocular dominance columns after brief monocular inactivation. Proc. Natl. Acad. Sci. U.S.A. 106, 12151–12155 (2009). 19581597 10.1073/pnas.0905092106 PMC2706271

[63] Uhlirova H.., The roadmap for estimation of cell-type-specific neuronal activity from non-invasive measurements. Philos. Trans. R. Soc. B Biol. Sci. 371, 20150356 (2016). 10.1098/rstb.2015.0356 PMC5003857 27574309

[64] Vanzetta I., Grinvald A., Coupling between neuronal activity and microcirculation: Implications for functional brain imaging. HFSP J. 2, 79–98 (2008). 19404475 10.2976/1.2889618 PMC2645573

[65] Demené C.., 4D microvascular imaging based on ultrafast Doppler tomography. Neuroimage 127, 472–483 (2016). 26555279 10.1016/j.neuroimage.2015.11.014

[66] Adams D. L., Sincich L. C., Horton J. C., Complete pattern of ocular dominance columns in human primary visual cortex. J. Neurosci. 27, 10391–10403 (2007). 17898211 10.1523/JNEUROSCI.2923-07.2007 PMC6673158

[67] Van Essen D. C., Newsome W. T., Maunsell J. H., The visual field representation in striate cortex of the macaque monkey: Asymmetries, anisotropies, and individual variability. Vision Res. 24, 429–448 (1984). 6740964 10.1016/0042-6989(84)90041-5

[68] Gesnik M., “Imagerie fonctionnelle par ultrasons de la rétine et des fonctions visuelles cérébrales,” PhD thesis, PSL Research University, Paris, France (2017).

[69] Blasdel G. G., Orientation selectivity, preference, and continuity in monkey striate cortex. J. Neurosci. 12, 3139–3161 (1992). 1322982 10.1523/JNEUROSCI.12-08-03139.1992 PMC6575662

[70] Grinvald A., Lieke E., Frostig R. D., Gilbert C. D., Wiesel T. N., Functional architecture of cortex revealed by optical imaging of intrinsic signals. Nature 324, 361–364 (1986). 3785405 10.1038/324361a0

[71] Ikezoe K., Mori Y., Kitamura K., Tamura H., Fujita I., Relationship between the local structure of orientation map and the strength of orientation tuning of neurons in monkey V1: A 2-photon calcium imaging study. J. Neurosci. 33, 16818–16827 (2013). 24133282 10.1523/JNEUROSCI.2209-13.2013 PMC6618528

[72] Dumoulin S. O.., In vivo evidence of functional and anatomical stripe-based subdivisions in human V2 and V3. Sci. Rep. 7, 733 (2017). 28389654 10.1038/s41598-017-00634-6 PMC5428808

[73] Lawrence S. J. D., Formisano E., Muckli L., de Lange F. P., Laminar fMRI: Applications for cognitive neuroscience. Neuroimage 197, 785–791 (2019). 28687519 10.1016/j.neuroimage.2017.07.004

[74] Zwanenburg J. J. M., Versluis M. J., Luijten P. R., Petridou N., Fast high resolution whole brain T2* weighted imaging using echo planar imaging at 7T. Neuroimage 56, 1902–1907 (2011). 21440070 10.1016/j.neuroimage.2011.03.046

[75] Klein B. P.., Cortical depth dependent population receptive field attraction by spatial attention in human V1. Neuroimage 176, 301–312 (2018). 29709626 10.1016/j.neuroimage.2018.04.055

[76] Boido D.., Mesoscopic and microscopic imaging of sensory responses in the same animal. Nat. Commun. 10, 1110 (2019). 30846689 10.1038/s41467-019-09082-4 PMC6405955

[77] Dow B. M., Snyder A. Z., Vautin R. G., Bauer R., Magnification factor and receptive field size in foveal striate cortex of the monkey. Exp. Brain Res. 44, 213–228 (1981). 7286109 10.1007/BF00237343

[78] Hubel D. H., Wiesel T. N., Sequence regularity and geometry of orientation columns in the monkey striate cortex. J. Comp. Neurol. 158, 267–293 (1974). 4436456 10.1002/cne.901580304

[79] Talbot S. A., Marshall W. H., Physiological studies on neural mechanisms of visual localization and discrimination. Am. J. Ophthalmol. 24, 1255–1264 (1941).

[80] Arcaro M. J., Livingstone M. S., Retinotopic organization of scene areas in macaque inferior temporal cortex. J. Neurosci. 37, 7373–7389 (2017). 28674177 10.1523/JNEUROSCI.0569-17.2017 PMC5546109

[81] Shung K. K., Sigelmann R. A., Reid J. M., Scattering of ultrasound by blood. IEEE Trans. Biomed. Eng. 23, 460–467 (1976). 977014 10.1109/tbme.1976.324604

[82] Macé E.., Functional ultrasound imaging of the brain: Theory and basic principles. IEEE Trans. Ultrason. Ferroelectr. Freq. Control 60, 492–506 (2013). 23475916 10.1109/TUFFC.2013.2592

[83] Rungta R. L., Chaigneau E., Osmanski B.-F., Charpak S., Vascular compartmentalization of functional hyperemia from the synapse to the pia. Neuron 99, 362–375.e4 (2018). 29937277 10.1016/j.neuron.2018.06.012 PMC6069674

[84] Hackett T. A.., Neurosurgical access to cortical areas in the lateral fissure of primates. J. Neurosci. Methods 141, 103–113 (2005). 15585294 10.1016/j.jneumeth.2004.06.001

[85] Goriely A.., Mechanics of the brain: Perspectives, challenges, and opportunities. Biomech. Model. Mechanobiol. 14, 931–965 (2015). 25716305 10.1007/s10237-015-0662-4 PMC4562999

[86] Errico C.., Transcranial functional ultrasound imaging of the brain using microbubble-enhanced ultrasensitive Doppler. Neuroimage 124, 752–761 (2016). 26416649 10.1016/j.neuroimage.2015.09.037 PMC4686564

[87] Boto E.., Moving magnetoencephalography towards real-world applications with a wearable system. Nature 555, 657–661 (2018). 29562238 10.1038/nature26147 PMC6063354

[88] Valero-Cabre A.., Frontal non-invasive neurostimulation modulates antisaccade preparation in non-human primates. PLoS One 7, e38674 (2012). 22701691 10.1371/journal.pone.0038674 PMC3368878

[89] Montaldo G., Tanter M., Bercoff J., Benech N., Fink M., Coherent plane-wave compounding for very high frame rate ultrasonography and transient elastography. IEEE Trans. Ultrason. Ferroelectr. Freq. Control 56, 489–506 (2009). 19411209 10.1109/TUFFC.2009.1067

[90] Blaize K., , Functional ultrasound imaging of deep visual cortex in awake nonhuman primates. Open Science Framework. https://osf.io/j8gqn/. Deposited 27 May 2020. 10.1073/pnas.1916787117 PMC7321983 32513717

