# Supplementary material for: Functional ultrasound imaging of deep visual cortex in awake nonhuman primates
Source: Proc Natl Acad Sci U S A. 2020 Jun 8;117(25):14453–63. doi: 10.1073/pnas.1916787117 (PMC7321983; doi:10.1073/pnas.1916787117)

PNAS
www.pnas.org

Supplementary Information for

Functional ultrasound imaging of deep visual cortex in awake nonhuman primates
Paste the full author list here

Kévin Blaize and Serge Picaud
Email: blaize.kevin@gmail.com and serge.picaud@inserm.fr

This PDF file includes:

Figures S1 to S5

www.pnas.org/cgi/doi/10.1073/pnas.1916787117

Figure S1. schematic representation of the different imaging planes with fUS imaging in monkey S (top and middle) and in monkey T (bottom). Left: 3D representation of the operculum of V1 (yellow) and V2 (blue) and the recording chamber (orange). Right: Atlas representation adapted from Saleem and Logothetis, 2015. uVM: upper vertical meridian; lVM: lower vertical meridian; HM: horizontal meridian.

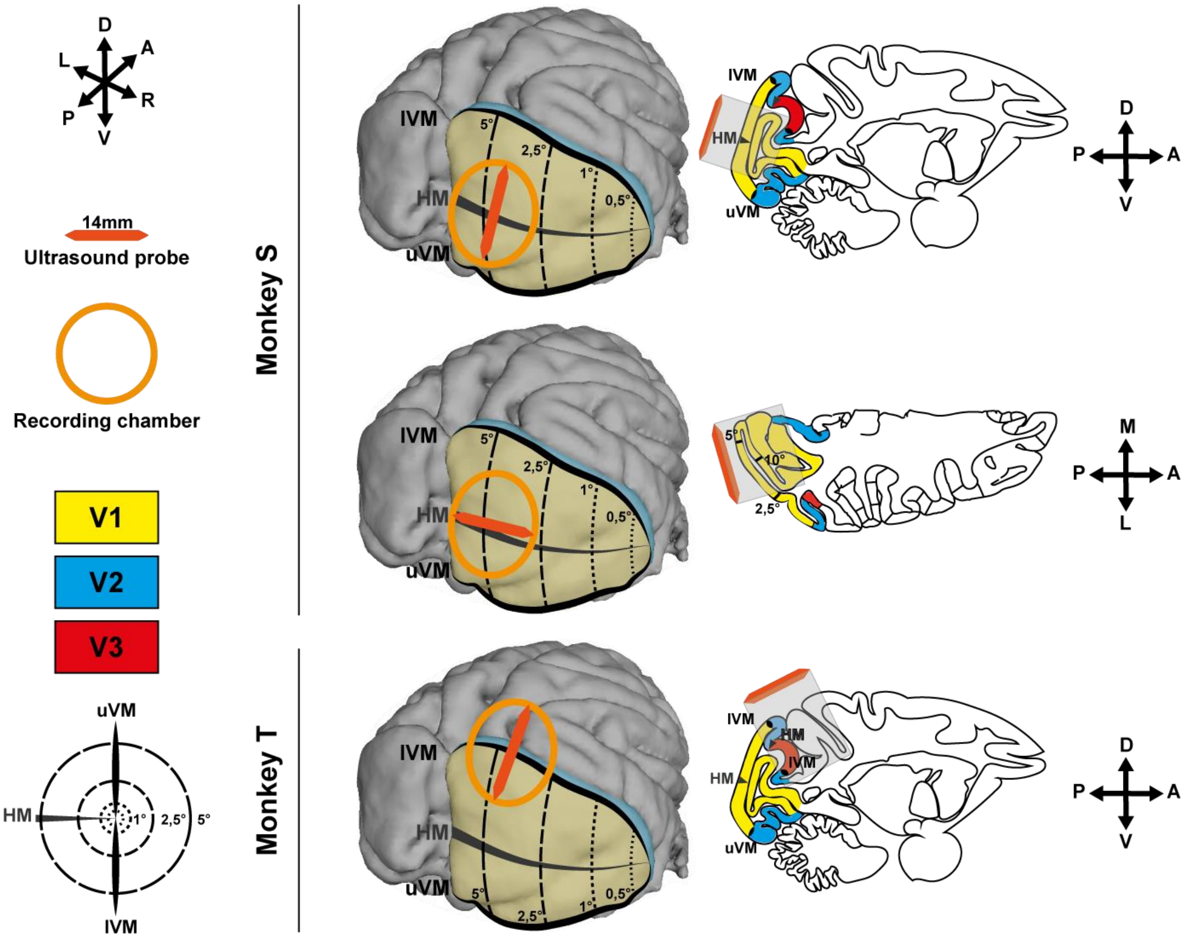

Figure S2. Control acquisition with no visual stimulation during a 2s fixation task. Upper: mean CBV signal evolution within the ROI represented in lower maps by the white dotted rectangles. Shaded area represents the SEM. Lower: Activation maps obtained according the number of averaged trials. Scale bar represents 2 mm. Black dotted line: calcarine sulcus.

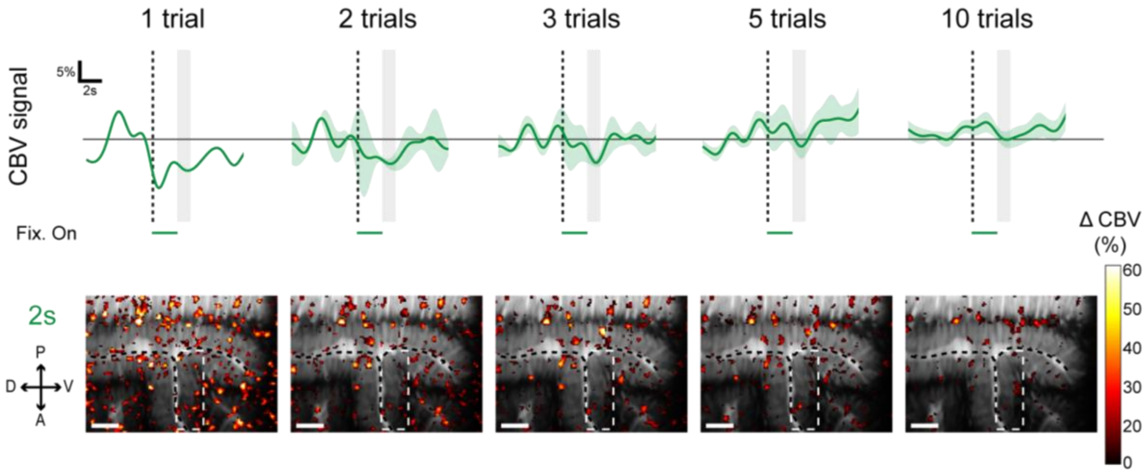

Figure S3. Reproducibility of the fUS imaging retinotopic maps. **a-d**) Different analyses on activation maps illustrated in Figure 2A. Only significant pixels are shown (threshold: $R^2 > 0.02$). (**a**) Maximal CBV response map for all eccentricity stimulations. (**b**) Retinotopic map obtained without extrapolation and smoothing. (**c**) Selectivity map represented with the Gaussian bandwidth. (**d**) Coefficients of determination map for a Gaussian curve fitting. **e-f**) Two eccentricity (**e**) and two angular (**f**) retinotopic maps obtained from four different sessions on four different days in monkey S. Note the slight differences in the functional maps and on the anatomical maps caused by slight differences in probe position on different acquisition days. Scale bars in A-F represent 2 mm. Black dotted line: calcarine sulcus.

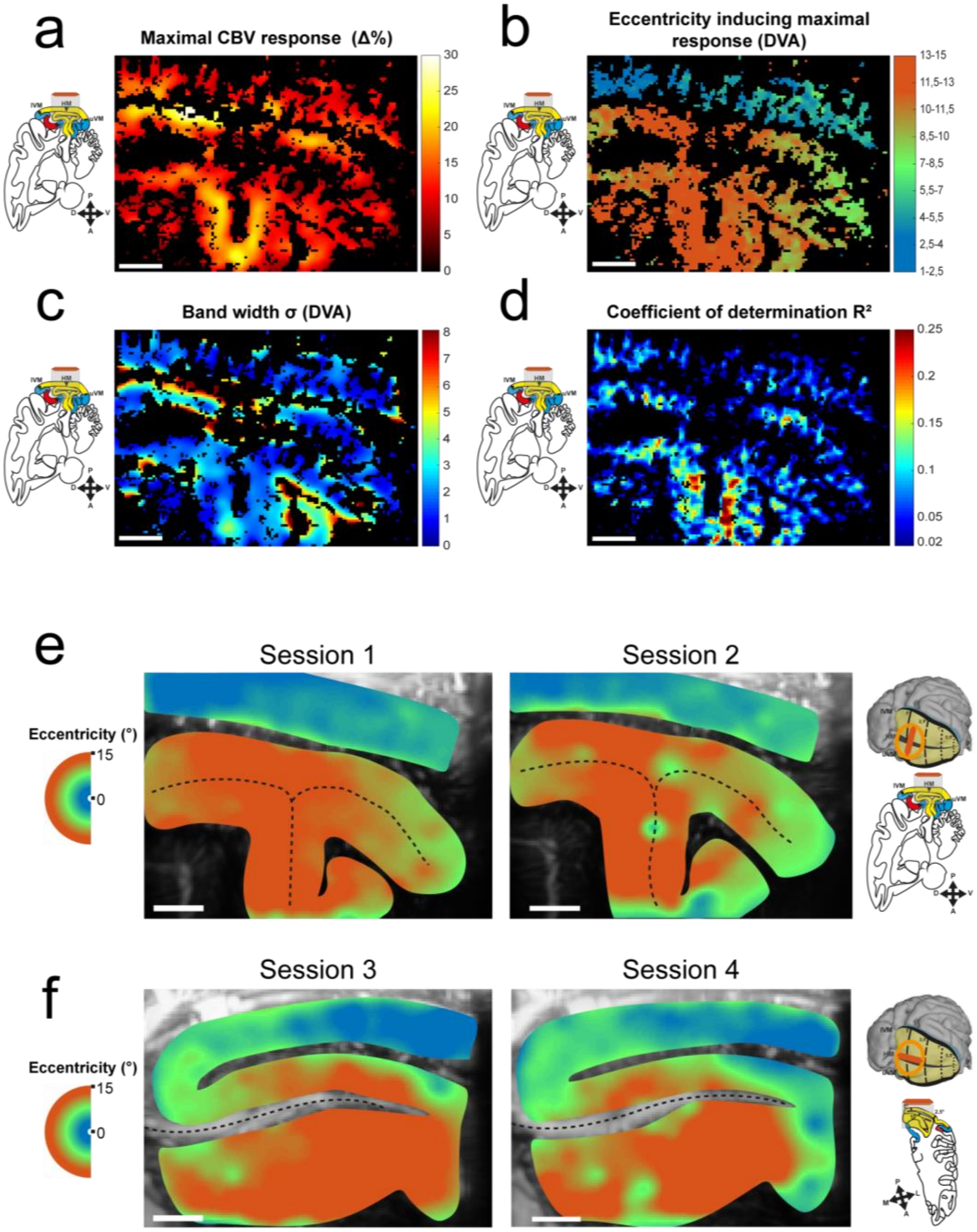

Figure S4. V1/V2 border localization with functional ultrasound maps. (**a**) Ocular Dominance map in monkey T (same as in Figure 3B. bottom). The bands have been highlighted by drawing the contours (black closed lines). Anatomical V1/V2 border location is represented by the black dashed line. OD bands are present in V1 and absent in V2. (**b**) CBV variations for lower vertical meridian stimulation (the stimulus is illustrated in the bottom right corner). The V1/V2 border confidence zone (vertical meridian activation) is located between the blue and the white arrows. The anatomical V1/V2 border is represented by the white dashed line. (**c**) First angular retinotopic map in monkey T (two sessions are pooled). (**d**) Second retinotopic map obtained in monkey T (Same as in Figure 2D. right panel). **c-d**) The V1/V2 border confidence zone is represented by the blue and the white arrows and the anatomical V1/V2 border by the black dashed line. We have reproduced the same arrows from panel b in panels c and d. Scale bars represent 2mm in all panels.

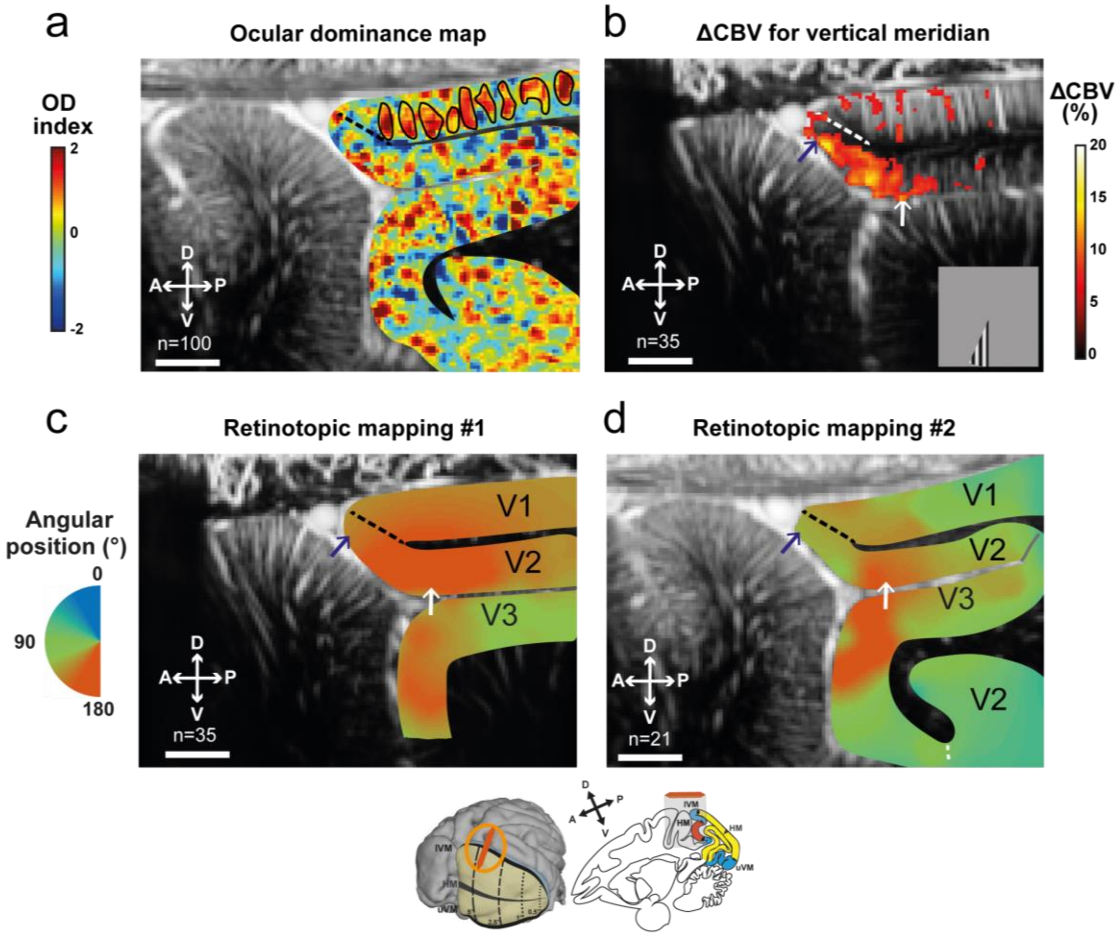

Figure S5. Analyses of OD maps. **a**) OD map (left) obtained for monkey S without cropping by subtracting ipsilateral blocks from contralateral blocks, with subsequent normalization. The corresponding control OD map was obtained for the same acquisition by shuffling ipsilateral and contralateral blocks so that even blocks were subtracted from odd blocks prior to normalization. Black dotted line: calcarine sulcus. **b**) Example of layer segmentation computation for ROI #1. Black dotted line: calcarine sulcus.

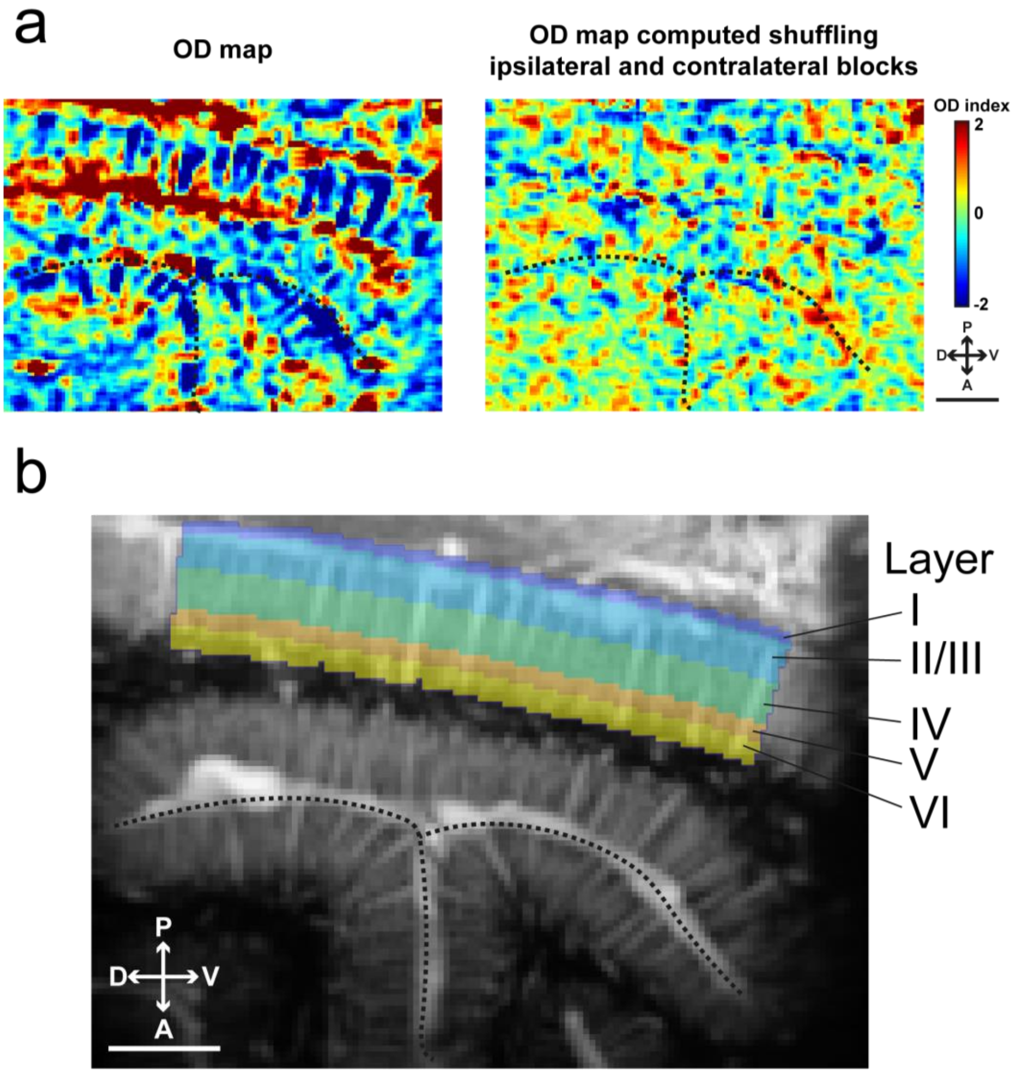

Supplement: Supplementary File [file pnas.1916787117.sapp.pdf]
